# Implementation and Evaluation of a Patient-Focused eHealth Intervention, My Kidneys My Health, in Primary Care and General Nephrology Clinics: Multimethods Study

**DOI:** 10.2196/71832

**Published:** 2025-08-29

**Authors:** Sabrina Jassemi, Dwight Sparkes, Maria Delgado, Lori Harwood, Aminu K Bello, Heather Beanlands, Meghan Elliott, Kerry McBrien, Sarah Gil, Sharon Straus, Brenda Hemmelgarn, Maoliosa Donald

**Affiliations:** 1 Department of Medicine, University of Calgary, 2500 University Drive NW, Calgary, AB, T2N 1N4, Canada, 1 (403) 220-5110; 2 Can-SOLVE CKD Network, Vancouver, BC, Canada; 3 London Health Sciences Centre, London, ON, Canada; 4 Department of Medicine, University of Alberta, Edmonton, AB, Canada; 5 Daphne Cockwell School of Nursing, Toronto Metropolitan University, Toronto, ON, Canada; 6 Department of Family Medicine, University of Calgary, Calgary, AB, Canada; 7 Department of Medicine, University of Toronto, Toronto, ON, Canada

**Keywords:** chronic kidney disease, self-management, implementation science, mhealth, nephrology care, patient-oriented research, electronic health, mobile health

## Abstract

**Background:**

Care for mild to moderate chronic kidney disease (CKD) entails self-management from patients and clinical support from primary care and nephrology. To address the gap in self-management resources, My Kidneys My Health was codeveloped to support patients with CKD. Health care providers play a critical role in the implementation of patient resources; however, there is a gap in understanding providers’ perspectives in this role.

**Objective:**

This study develops and evaluates strategies to implement My Kidneys My Health into routine primary care and general nephrology clinical care.

**Methods:**

Health care providers working in Alberta, Canada, who support patients with CKD were invited to participate in our multistep study, guided by the Quality Implementation Framework. In step 1, we followed qualitative descriptive methodology to identify barriers and enablers to implementation using a directed content analysis and a deductive coding approach. Participants were invited to complete semistructured interviews from October 2021 to May 2022. In step 2, we identified, prioritized, codeveloped, and launched implementation strategies based on step 1 results using behavior change theory. Participants were invited to use the materials during the implementation period (May to October 2022). Website engagement was tracked through Google Analytics and document distribution tracking. In step 3, we conducted follow-up interviews with participants (October to December 2022) to evaluate implementation based on the Reach, Effectiveness, Adoption, Implementation, and Maintenance framework, following the same qualitative approach as step 1. Effectiveness was out of the scope of this study.

**Results:**

A total of 16 health care providers participated in step 1 qualitative interviews (8 from nephrology clinics and 5 from primary care or nonambulatory care). Participants shared an individual-level readiness and interest in sharing My Kidneys My Health with their patients. The key barriers to implementation included awareness, memory, time, motivation, and innovation accessibility. Implementation strategies were co-designed and implemented by step 1 participants (ie, educational sessions and materials, reminders, and implementation coaching). Notably, 9 health care providers participated in step 3 qualitative interviews. Participants shared their approach to tailoring implementation based on their patients and integrating the resource into their current practices. The resources developed were highly used by participants, with positive feedback on their usability and accessibility. Participants expressed motivation to continue sharing My Kidneys My Health*;* however, awareness and accessibility require further adaptations that can improve sustainability of implementation. Our rigorous approach allowed us to address behavior change and sustainability of implementation of My Kidneys My Health, as well as identify appropriate and tailored implementation strategies.

**Conclusions:**

There is a readiness to implement self-management supports for patients with early-stage CKD. A theory-informed approach and strategic implementation strategies can support sustainability.

## Introduction

### Background

Care for chronic kidney disease (CKD), a progressive disease, is complex, and it entails not only management of CKD but also attending to other chronic conditions, such as diabetes and cardiovascular disease [1]. People living with CKD are mainly managed in primary care settings [2,3]. However, they also require care from specialists (eg, nephrologists) and allied health providers (eg, renal dietitians and social workers), who are often only accessible once their conditions worsen [4-6]. This results in patients navigating the complexity of CKD and its treatment, often with minimal guidance from health care providers. To combat this, patients are often required to become actively engaged in self-management strategies, which have been shown to slow the progression of CKD [7]. This entails independent monitoring of their diet, exercise, and medication management, often without structured supports or practitioners.

Patients and health care providers have called for interventions to address self-management needs to delay CKD progression and improve quality of life [8-13]. The self-management interventions that are currently available predominantly focus on later-stage CKD (eg, kidney care multidisciplinary teams) and were designed without patient input [9,14-16]. To address this gap, we developed the My Kidneys My Health website [17] for patients with mild to moderate CKD as part of a larger multiphase research program, including patients, caregivers, health care providers, researchers, and policy makers (Canadians Seeking Solutions and Innovations to Overcome Chronic Kidney Disease [Can-SOLVE CKD] Network) [13,18].

My Kidneys My Health (Tactica Interactive) [17] is a patient-facing tool co-designed with patients and caregivers through a user-centered design process specifically to provide support for their self-management of early-stage CKD [13]. We undertook a theory-informed, person-centered approach guided by the Knowledge-to-Action Framework [19] to develop an eHealth tool that is concordant with patients’ values, needs, and preferences for CKD self-management support. This website supports self-management by “informing” (chronic kidney disease–related information), “activating” (prompts or tools to encourage action to manage chronic kidney disease and enhance quality of life), and “collaborating” (links or tools that lead to interaction and engagement with health care providers and peers) [13,20]. Prior to the launch of My Kidneys My Health in March 2021, the website was tested and users found it to hold credible information that was user-friendly and particularly relevant for patients who are newly diagnosed with CKD [21].

Health care providers play a critical role as collaborative partners to support self-management for patients with mild to moderate CKD, including the provision of patient-facing educational resources, such as My Kidneys My Health. While there is foundational work looking at elements for health care providers to provide face-to-face self-management support strategies [22], there is a gap in knowledge and understanding of implementing a patient self-management eHealth tool in the clinical care setting to enhance patient self-management.

### Objective

The aim of this multimethods study was to rigorously develop and evaluate strategies for implementing the My Kidneys My Health website into routine clinical care in primary and general nephrology clinic settings by health care providers in the province of Alberta, Canada.

## Methods

### Overview

**Figure 1.**
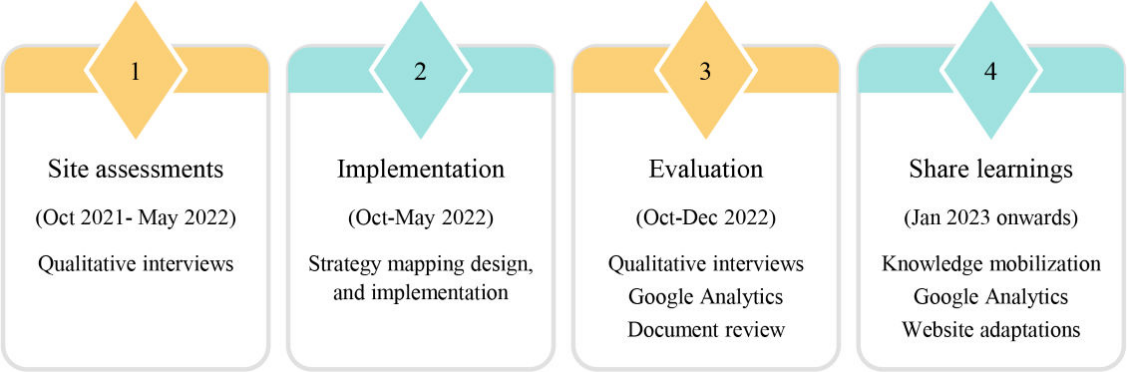
Quality Improvement Framework guiding implementation and sustainability of My Kidneys My Health.

We conducted a multistep study, guided by the Quality Implementation Framework [23], to develop and evaluate the implementation plan of My Kidneys My Health based on behavior change theory through qualitative and quantitative methodologies. Four steps were completed (as shown in Figure 1): (1) site assessments, (2) implementation, and (3) evaluation, with the ongoing application and (4) dissemination of lessons learned. An evaluation plan and logic model were developed to guide the planning and execution of these steps (Multimedia Appendix 1). Our qualitative methods are reported using the COREQ (Consolidated Criteria for Reporting Qualitative Research) guidelines [24]. Our research team included 5 patient partners with lived experience with CKD, who supported this work from the inception of the larger multiphased project [9-13,16,25-27]. Specific to this study, they provided feedback on research materials, implementation strategies, and informed interpretation of results.

### Participants and Setting

At the time of the study, Alberta’s provincial delivery system, Alberta Health Services, was divided into 5 health zones (North, Edmonton, Central, Calgary, and South) that serve remote, rural, and urban jurisdictions and support care to marginalized groups. Within these zones, Alberta Kidney Care North and South provide clinical services to people followed by nephrologists and multidisciplinary care to individuals with advanced CKD. Most primary care providers provide community care supported by 39 Primary Care Networks. Alberta Kidney Care and primary care clinics vary in their size, remuneration, available resources, and staff composition (eg, access to multidisciplinary team). In Alberta, the COVID-19 restrictions were enacted from March 2020 to June 2022. As a result, during our study recruitment and data collection period, there were fewer in-person appointments, and as a result, less screening was conducted to identify patients with CKD.

Using a purposive sampling strategy, we invited health care providers (eg, primary care physicians, nephrologists, and allied health care professionals), as well as clinic administrators who oversee site operations with key decision-making roles, who worked in Alberta, Canada. To maintain the relevance of the study results, we limited our recruitment to specifically invite health care providers, who provided direct clinical care and education to patients with mild to moderate CKD (ie, patients who are not on dialysis or who have not received a kidney transplant). We also aimed to recruit participants with varied experience using My Kidneys My Health. Recruitment information was circulated through local Primary Care Networks, as well as Alberta Kidney Care North and South. We aimed to recruit 25-30 interview participants for steps 1 and 3, based on previous similar studies [28,29]. Potential participants were invited to participate in all 3 steps of this study.

### Step 1: Site Assessments

We used a qualitative descriptive methodology [24,25], where we conducted semistructured telephone interviews with participants between October 2021 and May 2022 to identify perceived barriers and enablers to the implementation of My Kidneys My Health in clinical practice. The semistructured interview guide (Multimedia Appendix 2) was designed to assess readiness [30] and potential sustainability of the intervention [31]. Telephone interviews were conducted by experienced qualitative interviewers (SJ identifies as a woman, has interest in kidney health research; MD, identifies as a woman, has a clinical background as a physical therapist and has expertise in mixed-methods), audio recorded, and transcribed verbatim by a trained transcriptionist, removing identifying information. Debrief meetings were held concurrently with data collection to discuss preliminary results from data collection and to identify when to end recruitment due to a saturation of results.

We completed a directed content analysis using a deductive coding approach [32]. Each transcript was entered into NVivo version 11 (QSR International Pty Ltd) 2016, and coders (SJ and MD) familiarized themselves with the data and independently and iteratively coded using a codebook developed prior to analysis, which remained unchanged throughout analysis. The codebook was based on the Capability Opportunity Motivation Behavior System Framework (COM-B) [33], which was further framed by the Consolidated Framework for Implementation Research (CFIR) [34,35] and the Theoretical Domains Framework (TDF) [36]. Consensus meetings were held iteratively to ensure consistency and refinement of our coding. Interrater reliability was monitored as a guide to discussion, and Cohen kappa coefficient results below 0.6 were reviewed [37]. From these codes, we created overarching categories of barriers and enablers used to inform the identification and creation of implementation strategies.

### Step 2: Implementation

**Figure 2.**
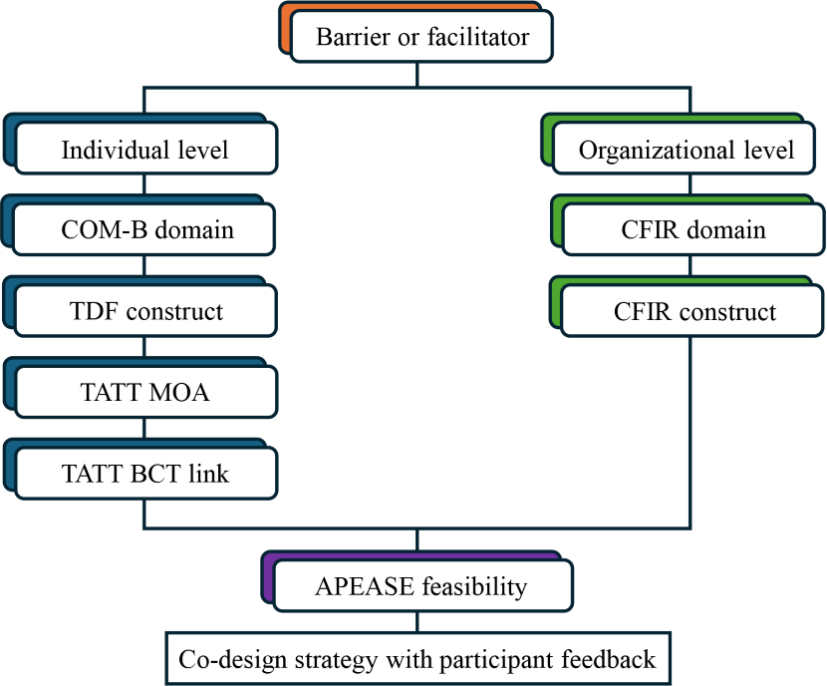
Step 2 guide to theory-informed strategy identification process based on barriers and facilitators Activity 1 barriers and facilitators. APEASE: acceptability, practicability, effectiveness, affordability, side effects, and equity tool; BCT: behavior change theory; CFIR: Consolidated Framework for Implementation Research; COM-B: Capability Opportunity Motivation Behavior System Framework; MOA: mechanism of action; TATT: Theory and Techniques Tool; TDF: Theoretical Domains Framework.

The barriers and enablers identified in step 1 were mapped to potential strategies and used to develop an implementation plan (Figure 2). The CFIR 2.0 provided a framework to inform appropriate strategies [35,38], and the Theory and Techniques Tool [39] was used to map the mechanism of action and behavior change techniques for specific approaches. Implementation strategies were then prioritized based on the acceptability, practicability, effectiveness, affordability, side effects, and equity (APEASE) tool [36] to identify feasible approaches that would meet the context of this study.

With the support of a graphic designer and feedback from our patient partners, strategies were developed using the action, actor, context, target, time (AACTT) framework [40] and evidence-based system for implementation supports [30]. In addition, we engaged a national CKD Clinic Network’s knowledge broker, as well as local health partner organizational networks in Alberta, iteratively throughout the development of strategies. Each strategy incorporated features that allowed us to track uptake (eg, individual QR codes that linked to Google Analytics, tracking sheets) to support the evaluation in step 3 through a quantitative approach using descriptive statistics. The implementation strategies were launched in July 2022 for participants from the step 1 site assessment interviews, facilitated by the study coordinator (SJ), and tracked throughout the implementation and evaluation period (May to December 2022). Though all Google Analytics could not be stratified by implementation strategy, select measures and a document distribution tracker were monitored throughout the implementation period to inform any trends in implementation (eg, uptake of tools, gaps to address, whether to print more materials), which were incorporated into step 3’s methodology and interpretation of results.

### Step 3: Evaluation

Following the implementation period, we conducted a process evaluation through qualitative interviews and document review as guided by our logic model and evaluation matrix. Website traffic and engagement data were extracted from Google Analytics to track the usage of the printed materials through their unique QR codes during the implementation period. The volume of materials shared with participants was documented (ie, the number of materials shared, when they were shared, and whom they were shared with) to supplement the Google Analytics data and inform the uptake of the materials by the providers and recipients. The implementation plan was iteratively informed by the feedback we received from participants, partners, and behaviors of website users via Google Analytics.

Our qualitative interviews and analysis followed the same qualitative descriptive methodology described in step 1. We recruited previous participants and used snowball recruitment for further participants who were exposed to the implementation strategies to mitigate the expected attrition due to environmental impacts (ie, changes in personnel and roles due to the COVID-19 pandemic and the changing landscape of health care in Alberta). From October to December 2022, we conducted semistructured telephone interviews to understand how My Kidneys My Health was implemented, based on the Reach, Effectiveness, Adoption, Implementation, and Maintenance (RE-AIM) framework (Multimedia Appendix 3). The RE-AIM framework is a planning and evaluation framework with 5 individual- and setting-level dimensions [41]. Effectiveness was beyond the scope of this evaluation, given the short length of the implementation period. The interview guide was also tailored based on Google Analytics results, informing probes about the strategies as well as remaining barriers and enablers. The deductive qualitative analysis was guided by the RE-AIM framework and combined with the quantitative results to gain a full picture of the implementation.

### Ethical Considerations

Ethics approval was obtained from the University of Calgary, Alberta (number REB21-0930). All study participants provided their informed consent orally for their participation in qualitative interviews and through implied actions for the demographic survey. Data was managed by the study team (SJ and MD) confidentially. Participants received a gift card of CAD $50 (US $36.21) for their participation in the step 1 interview, as well as the step 3 interview.

## Results

### Step 1: Site Assessments

Sixteen health care providers participated in telephone interviews (average length of 33 min). Three participants did not complete the demographic questionnaire and were not included in the participant characteristics. Participants included nephrologists (n=5, 39%), pharmacists (n=3, 23%), and 2 primary care physicians (n=2, 15%; Table 1). Participants were largely working in general nephrology clinics (n=6, 46%) and primary care (n=5, 38%), from large urban centers (n=11, 84%). Most participants were female (n=8, 61%). Recruitment for step 1 was concluded once we met data saturation. Reasons for nonparticipation included self-reported lack of opportunity to share the tool, given the frequency that they see patients with mild to moderate CKD (eg, infrequent visits, primarily see patients in dialysis clinics or who have received transplants). Barriers and enablers were categorized under the COM-B domains, highlighting a readiness for participants to implement the website into practice (Table 2).

**Table 1.** Step 1 site assessment interviews: participant demographics characteristics (N=14)^a^

Participant characteristics	Value, n (%)
Current primary role	
Nephrologist	5 (39)
Pharmacist	3 (23)
Primary care physician	2 (15)
Nurse	2 (15)
Nurse practitioner	1 (8)
Clinical setting	
General nephrology clinic	6 (46)
Primary care practice	5 (38)
Specialty nephrology clinic (eg, glomerular nephritis)	1 (8)
Other (nonambulatory care)	1 (8)
Geographical location	
Large urban centers (>100,000)	11 (84)
Medium rural population centers (30,000 to 99,000)	1 (8)
Small rural population centers (<30,000)	1 (8)
Age (years)	
Younger than 40	6 (46)
Older than 40	6 (46)
Prefer not to answer	1 (8)
Current employment status	
Full-time	9 (69)
Part-time	4 (31)
Team members within practice (select all that apply)	
Nurse	11 (84)
Dietitian	11 (84)
Social worker	10 (77)
Pharmacist	9 (69)
Administrator	8 (62)
Nephrologist	7 (54)
Primary care physician	5 (38)
Nurse practitioner	4 (31)
Gender	
Woman	8 (62)
Man	4 (31)
Prefer not to answer	1 (8)
Years in clinical practice	
0-5	2 (15)
6-10	3 (23)
11-15	3 (23)
more than 16	5 (38)

a Step 1: 3 out of 16 respondents did not complete the demographic questionnaire and were not included in the demographic data.

**Table 2.** Step 1 site assessment results: description of barriers and enablers to implementing My Kidneys My Health in clinical practice.

Category and domain		Description	Quotations
Barriers			
Capability		Lack of awareness (website and content)	“I kind of know the gist of the layout, but I’m not at all familiar with the functionality and all the features of it.”(HCP5)
Opportunity		Concerns around memory and habit forming (competing priorities and resources)	“I think it’s not that people don’t want to use it; it’s just getting them to remember about it.” (HCP2)
Motivation		Perceived patient accessibility concerns (language, internet, technology, and health literacy) Clinic’s environmental context limits ability to share resources	“Our average age of our patients is 72 and while the website seems to be relatively accessible, there should be a paper equivalent to be available also.” (HCP11) “Bringing in like a computer or laptop and then having to like worry about sanitizing it especially if they are on any kind of precautions while they are in hospital. So, using the tool for teaching can be a little bit trickier in hospital right now…” (HCP4)
Facilitators			
Capability		Intervention fits existing skills (patient education), abilities (technical), and practices	“One of our main roles… is to provide patient education and support to disease self-management and so I think it fits well for that because we do in addition to providing education, provide resources and so this is a good online resource” (HCP1)
Opportunity		Tailored use of intervention saves time in clinic (supplements education)	“Instead of spending like 30 minutes with them, I can spend 10 minutes and then tell them go and read this website and then we chat again next time... That would simplify my practice, save my time” (HCP14)
Motivation		Intervention fits values (accessible, credible, and useful) and practices (sharing resources, personalizing care, and supports education) Clinic readiness (existing culture for change, leadership advocacy, and knowledge sharing between trusted coworkers)	“It definitely fits into my values and norms because I feel it provides a lot of really great information and it just gives that extra information for that patient, so many are wanting to find stuff online, so it gives a credible resource to go to.” (HCP6) “Typically, if there is a really good resource that comes across somebody’s table, and they want to share it… typically the managers will review it and just make sure kind of it lines up with national guidelines and it’s evidence based.” (HCP3)

#### Capability

Participant awareness of My Kidneys My Health presented as a main barrier to implementation. Prior to the study, most participants were not aware of the website. Those who were aware had limited knowledge of website content and features and shared uncertainty in how to present it to patients. Once participants were made aware of My Kidneys My Health, they shared that it aligned with their perceptions of their clinical roles, abilities, and values with regard to empowering patient self-management and sharing educational materials, including digital resources. Despite participant motivation to share the website, some participants were concerned about their capability to implement due to competing resources (ie, other patient educational materials), remembering to share My Kidneys My Health in clinical practice, and the effort required to share the resource given their busy workload.

#### Opportunity

Conversely, some participants believed that sharing My Kidneys My Health with patients would not increase time in appointments and may in fact simplify their educational component of appointments with patients as a supplementary tool, thereby saving time. These participants planned to personalize their integration within the clinic visit by sharing the relevant sections of the website to meet the specific needs of patients based on CKD status or questions identified in conversation. Participants identified that there were existing multimodal communication opportunities with patients (eg, letters, emails, verbal, and physical demonstration), as well as flexible opportunities (eg, prior to, during, or after appointments). Those who identified time as a barrier to implementation defined their approach as reviewing the website in detail and indicated that including a new resource would require additional efforts. Time was identified as a barrier by physician participants only; allied health providers did not list this as a concern. Many allied health providers and physician participants shared that they have the time to incorporate the intervention into their practice.

#### Motivation

Participants reported that the relevance and reliability of content, personalization of information, and formatting of the website aligned with their clinical values for caring for their patients. They also saw alignment with perceived patient needs for accessible information that would support patient self-efficacy in their self-management. In addition, the influence of participants’ inner setting was considered. Specifically, trusted colleagues and leadership supported participant uptake of resources; however, the impact of the physical context of their appointments posed some concerns (eg, access to a computer, telephone appointments). The influence of outer setting barriers posed a potential challenge to motivation, specifically related to competing resources and participants’ perception of patient comfort with self-management. Though participants found the website to be straightforward and easy to navigate, accessibility of the website was questioned based on patient ability or access. Specifically, that uptake would require access to the internet, access to an electronic device, the ability to navigate the website, and interpretation of the information. Furthermore, participants identified that patients may require additional language translation options to meet their needs. Finally, concern was raised regarding whether patients would use the website after being introduced to it.

### Step 2: Implementation

The implementation period included the mapping, prioritizing, design, and launch of the implementation strategies identified based on step 1 results. In May 2022, the barriers and enablers were mapped onto individual and external setting implementation strategies, then prioritized based on the feasibility measured by the APEASE framework [36]. Five barriers and enablers from step 1 and associated strategies that were prioritized are awareness, memory, time, motivation, and innovation accessibility (Table 3). Participant recommendations and accommodating enablers were considered when planning the implementation strategies. With the support of a graphic designer and review by patient partners, implementation strategies were tailored based on the participant needs and the prioritized barriers. The distribution of these implementation strategies was tracked using Google Analytics and documentation of resource distributions and educational outreach. Unique QR codes were incorporated into each material, which allowed us to track how many website users came from each resource through Google Analytics.

**Table 3.** Step 2 implementation strategy results: description of prioritized barriers and associated theory-informed implementation strategies.

Top barriers and APEASE^a^ feasibility	Domain and construct	Mechanism of action	Interventions and behavior change links	Existing facilitators
Memory (medium)	Capability, Psychological Enablement and Training	Memory, attention, and decision processes	Reminders, prompts, and cues Commitment	Accessible reminders into practice
Awareness (high)	Capability, Psychological; Inner Setting Access to Knowledge and Information	—^b^	Distribute educational materials Instruction Information about health consequences, social environment restructuring	Existing capacity building opportunities
Time (medium)	Opportunity, Physical Enablement	Environmental context and resources	Problem-solving Social support (practical) Restructuring physical environment	Leadership support, patient touchpoint opportunities in appointments
Motivation (high)	Education Motivation, reflective	Motivation Discrepancy: current behavior vs goals	Goal setting (outcome) and review Incentive (outcome) Educational materials	Adaptability and benefits of tool, influence of trusted coworkers, local opinion leaders
Accessibility (low to high)	Innovation Innovation Adaptability Innovation Design	Beliefs about consequences	Develop educational materials Identify early adopters Conduct local needs assessment Problem-solving	Familiarity of website, collaborative care models, personalizing implementation to audience

a APEASE: acceptability, practicability, effectiveness, affordability, side effects, and equity.

b Not applicable.

First, we completed 5 educational sessions tailored to various clinical audiences (ie, local and provincial renal and primary care groups) and 6 academic research activities (eg, local, national, and international presentations; a paper) to address awareness, time concerns, and motivation to share My Kidneys My Health. For example, a presentation was provided to a Primary Care Network in Alberta, which included primary care providers and allied health professionals who care for patients with CKD. The session outlined the evidence-based and patient co-design approach of the website, as well as the content, features, and flow of information on the website. To mitigate time and motivational barriers, we provided case examples of how the website could be shared with a patients (eg, as an educational tool for patients who were recently diagnosed with CKD or who had a recent health change), demonstrating opportunities to integrate the website within their existing clinic workflow. A detailed how-to implementation guide and a one-page summary of My Kidneys My Health were also developed to support provider knowledge of the website, the content available, and how they could incorporate it into their practice. These could be shared among providers and allowed us to raise awareness for providers who could not attend education sessions.

In addition, we developed a multifaceted reminder system for providers, which included monthly emails from the implementation coach (SJ, a member of the research team, to support participants with implementation) and visual cues built into existing routines. A bundle of educational materials was sent to participants, including posters and postcards with information about the website, as well as one-page educational materials about CKD for patients. These materials were also sent electronically for participants to share electronically or in print for patients. Participants were recommended to place these posters and postcards in their clinic as a visual cue to share the website or to prompt patients and caregivers to learn more about My Kidneys My Health. In addition, the website was shared through external partners in their patient dissemination materials (eg, the QR code was embedded in the Blood Tribe Department of Health materials), as well as newsletters and web postings (eg, through the Canadian Primary Care Sentinel Surveillance Network).

### Step 3: Evaluation

In total, 9 health care providers participated in a postimplementation telephone interview (average length of 27 min). Overall, 6 out of the 14 participants from step 1 did not participate in step 3 due to a change in role, lack of availability, or did not specify. As such, 3 new participants were recruited through snowballing, all of whom had exposure to an implementation strategy from our study. Sampling continued until no new concepts related to implementation experiences were identified in interviews. As with step 1, participants were mainly female, nephrologists or allied health care providers, from general nephrology and primary care clinics in large urban centers (Table 4). The implementation process was evaluated based on the RE-AIM framework, triangulating the interview data with material distribution tracking and Google Analytics data as a signal regarding implementation impacts (Table 5). Evaluating the effectiveness of the website (eg, change in self-management behaviors) was beyond the scope of this study.

**Table 4.** Step 3 evaluation interview: participant demographics characteristics (n=8)^a^.

Participant characteristics	Value, n (%)
Current primary role	
Nephrologist	3 (39)
Pharmacist	2 (25)
Nurse	1 (12)
Nurse practitioner	1 (12)
Decision maker	1 (12)
Clinical setting	
General nephrology clinic	4 (50)
Primary care practice	2 (25)
Other (nonambulatory care)	2 (25)
Geographical location	
Large urban centers (>100,000)	7 (88)
Small rural population centers (<30,000)	1 (12)
Age (years)	
Younger than 40	4 (50)
Older than 40	4 (50)
Current employment status	
Full-time	6 (75)
Part-time	2 (25)
Team members within practice (select all that apply)	
Nurse	7 (88)
Dietitian	7 (88)
Social worker	6 (75)
Administrator	4 (50)
Pharmacist	4 (50)
Nephrologist	4 (50)
Primary care physician	2 (25)
Nurse practitioner	2 (25)
Other	2 (25)
Gender	
Woman	7 (88)
Man	1 (12)
Years in clinical practice?	
0 -5	2 (25)
6-10	2 (25)
11-15	4 (50)
More than 16	0 (0)

a Step 3: 1 respondent of 9 did not complete demographic questionnaire, and was not included in the demographic data.

**Table 5.** Step 3 evaluation results: Reach, Effectiveness, Adoption, Implementation, and Maintenance domain definitions and results.

Domain	Description	Results	Quotations
Reach	The number and types of providers who implemented *My Kidneys My Health* into practice, description of recipients	7 of 9 completed implementation Provided education to patients (nephrologists, chronic disease educators, and pharmacists) Recipients: patients and caregivers with technical ability to use website, high self-efficacy, needed information (eg, new patients, change in health status) 2 of 9 did not complete implementation Acute care settings, role scope	“I’m sometimes hesitant when there’s people that I know who are like, oh, no I don’t go on the internet very often. I don’t really know how to navigate.” (HCP8)
Effectiveness**:** Out of scope.			
Adoption	How the implementation of *My Kidneys My Health* was tailored to patient characteristics and context, including description of overall implementation strategies used.	Tailoring completed based on patient characteristics and readiness: age, technical capability, and health status Impacts to implementation Timing and depth of exposure to intervention Printed and mailed more materials (total: 950 postcards, 25 posters, and 400 one-pagers)	“The ones that are like really comfortable, they’ll bring up the website right in clinic as opposed to holding onto that postcard and you know, wanting to consult with their middle-aged daughter about like how to get to the website and stuff.” (HCP9)
Implementation	How providers shared *My Kidneys My Health* in their clinical practice, including feedback on implementation strategies.	Description of implementation: Demonstrated website in appointment, emailed resources postappointment, described website and shared take-home resources; follow-up with patients on next visit to answer questions Shared with colleagues at formal (clinical rounds) and informal meetings Feedback on strategies: Barriers: clinic set-up, unsure if patients follow their direction, memory, habit Enablers: supports education, electronic and printed resources, reminders (emails and presentations	“As I’m looking at patient, I see the poster out of the corner of my eye it kind of reminds me to talk about it if I might have otherwise forgotten. And, then again having that presentation a couple of weeks of ago just kind of brought the whole website and the whole program to the forefront of my brain again” (HCP5)
Maintenance	Factors that may support or inhibit continued implementation of *My Kidneys My Health,* as well as suggestions based on sustainable strategies.	What worked well: Empowers patient/caregiver Supports education, enhances discussion with patient Saves time for clinicians Strategies to support sustainability: Create more printable materials Develop continuous reminders system Further integrate into existing practice (eg, waiting room, education for staff)	“It’s almost like you pay, you invest that time at the beginning…you end up saving yourself time in the long run, by empowering them with information.” (HCP7)

#### Reach

Overall, 7/9 participants implemented My Kidneys My Health, working in nephrology clinics, chronic disease management, or related to diabetes care optimization. Two participants did not implement the website into their clinical care routine due to lack of awareness and the setting where they provided care (acute care unit). Among those who implemented it, My Kidneys My Health was shared with patients and caregivers of varying ages and CKD status, including high-risk and newly diagnosed patients. Participants believed that certain clinical roles would be very impactful in implementing the resource, including chronic disease nurses and diabetes educators, as well as clinic staff who are the first points of contact with patients, such as medical office assistants.

#### Adoption

Participants tailored their implementation based on patient access and comfort with computers. For example, if a patient was less comfortable with using the website, the health care provider oriented them to the relevant website pages, provided paper-based information, or shared the website with a caregiver. Participants tailored implementation based on patient age or disease status but felt that ultimately the information should be shared with these patients to meet their self-management needs. Of paper-based printable resources, 950 postcards, 25 posters, and 400 one-pagers were distributed by the implementation participants. Individualized QR codes proved to be an effective method of tracking dissemination, as over 118 new users were brought to the website directly from our printable resources based on data from Google Analytics. Based on QR code tracking, the postcards brought in the most website users directly. We also observed a 10% increase in website users’ postintervention through Google Analytics data, compared to the previous period.

#### Implementation

Most participants shared the resource during clinical appointments in person or online, by Zoom (Zoom Communications) or telephone. Barriers to implementation included the clinic’s appointment room set-up (eg, lack of computers, positioning of the monitor, and size of the room), impacting their overall time for education in appointments. Printable materials presented an alternative option to support patient education during or after appointment times when the website was not accessible. Participants found reminders of the resources and website to be helpful mechanisms of action to support implementation, including a presentation to a multidisciplinary Primary Care Network, which reviewed website content and interactive features. A peak in website users was identified in Google Analytics on the day of this presentation and continued for the remainder of the week, showcasing how this strategy increased awareness and usage of the website. Webpages related to food and diet continued to be the most visited pages.

#### Maintenance

All participants shared that they would like to continue implementation of My Kidneys My Health into clinical practice by sharing it with their colleagues and patients. Providers found the website usable with clear and comprehensive self-management information for patients. Motivating factors to continue sharing the website included its design as well as the potential for impacts on patient outcomes. As predicted by step 1 participants, the website was believed to be a beneficial educational tool for patients to use outside of appointments, and participants felt it empowered patients to manage their own health as well as ask questions. One participant was concerned about patient uptake of resources in general, including this website, while others believed that navigating the website with the patient would increase the uptake of the website. In addition, participants also provided suggestions to improve the sustainability of the implementation. These included creating more paper-based, printable materials; periodic and multimodal dissemination; and highlighting aspects of the website, such as the My Questions List feature. Continuous dissemination and reminders appeared to be effective in addressing capability and awareness of health care providers, as we also continue to adapt implementation strategies based on the feedback received.

Long-term plans were also developed to address accessibility concerns related to My Kidneys My Health. The positive feedback received on the printable materials spurred further creation of printable materials that summarize website content and will be translated into other languages besides English, addressing concerns regarding accessibility of the website and language translation. This included creating additional printed materials, translating educational materials into additional languages, and conducting a needs assessment with diverse populations (eg, Indigenous communities in Alberta) to identify culturally relevant adaptations to the website. These strategies are to be developed based on the results from the evaluation phase and will be housed on the website for easy access for patients and health care providers.

## Discussion

### Principal Findings

**Figure 3.**
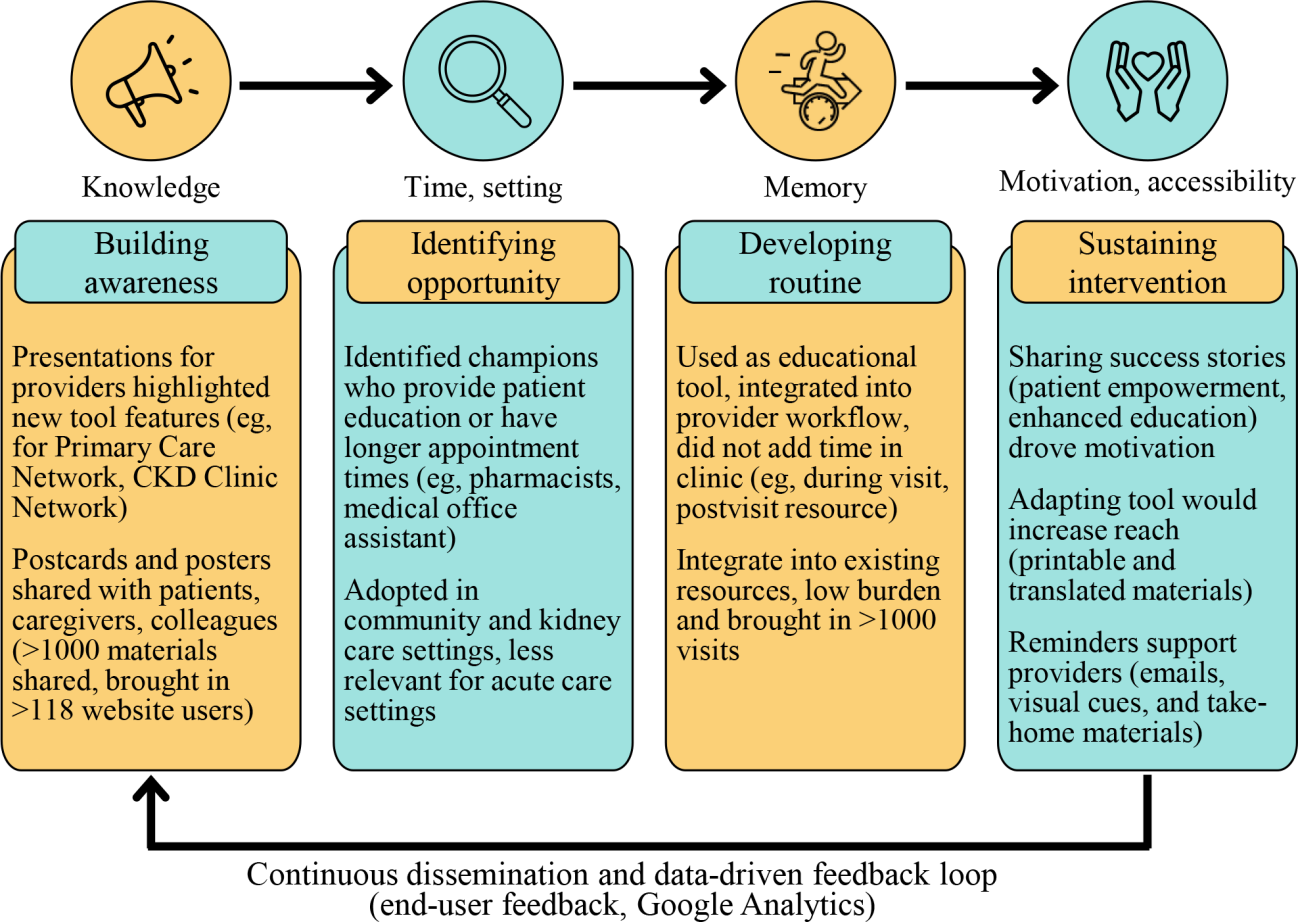
Implementation cycle: summary of strategies to address barriers and data-driven feedback loop. CKD: chronic kidney disease.

Following the Quality Implementation Framework [23], we systematically assessed, planned, and evaluated the implementation of a patient-facing eHealth tool, My Kidneys My Health*,* into primary care and nephrology settings in Alberta. This rigorous approach led us to addressing behavior change and sustainability of implementation of the tool itself, as well as identifying appropriate and tailored implementation strategies (Figure 3). We identified that there is readiness to implement an eHealth tool to support patient education and self-management, specifically for early stages of CKD. Further, health care providers can be an effective method of disseminating a patient-facing tool in clinical practice.

Delivering educational sessions was more successful in engaging health care teams and building awareness to implement the tool than passive dissemination strategies. We were also able to address various barriers through case studies in our presentations. Participants shared enthusiastic support of the printable materials, which were shared with colleagues, patients, and caregivers. Multimodal strategies enabled providers to tailor their implementation based on the patient needs. Consistent with our findings, continuous dissemination and iterative strategies can be effective in implementation [42]. However, further embedding reminders and ease of access to My Kidneys My Health materials would be integral to sustaining this intervention further. Google Analytics tracking through unique QR codes continues to be a successful metric in monitoring the uptake of implementation strategies.

### Implications for Research and Clinical Practice

While digital chronic illness self-management tools for patients have been studied in clinical settings [43,44], there has not been the implementation of such a tool for CKD where patients may be interacting with health care providers in specialty and primary care settings. Most studies that examine patient portals found that the intervention was not delivered by an individual care provider or administrator [45], which could limit the uptake of such tools by patients who may need encouragement and support to access these resources. Our study prospectively assessed provider readiness prior to implementation and continued to follow providers post implementation. Dissemination and implementation efforts can help bridge the gap between how patient-facing eHealth tools can be implemented into routine clinical practice within primary and specialty care settings. Through incorporating patient, caregiver, and organizational partner perspectives, we were able to tailor implementation strategies based on barriers and enablers, using evidence-based behavior change frameworks. To support implementation, continuous dissemination, development of offline resources, and environmental supports were effective strategies. Iterative feedback allowed us to further refine or innovate strategies, including adaptations to the intervention itself. For example, a web page dedicated to resources was developed to house printable materials, videos, and resources in one location for all website users.

The role of health care providers in supporting patients in self-managing their CKD cannot be underestimated. Traditional models of the provider-patient relationship and how education is provided must be revisited. Patients are feeling more empowered and more inclined to be involved in their health and health decision-making [43]. Health care providers have had to adapt to not being an intermediary (ie, all knowing of information and passing it on) but taking on a role of an apomediary (ie, directing the patient to high-quality information and services) [46]. My Kidneys My Health provides a solution that supports information sharing during a clinical encounter and can be tailored to each patient, potentially strengthening the provider-patient relationship [47].

### Strengths and Limitations

Strengths of this study include the rigorous application of implementation frameworks and theories from the preimplementation phase to the evaluation phase. However, there are some limitations to consider. Recruitment of health care providers, specifically in primary care, was affected due to the impacts of COVID-19 and the changing landscape of health care in Alberta, which posed challenges to staffing and burden of care. As a result, limitations included selection bias and a small number of participants recruited in step 1, a pool that we recruited from for step 3. Therefore, we recruited participants in step 3 who did not participate in step 1 but who did engage in implementation activities and could provide insights into their implementation experience. Despite this challenge, we were able to interview health care providers across a variety of roles and clinical settings.

In addition, the evaluation could not directly correlate the effectiveness of implementation strategies to website user data from Google Analytics, given the complexity of this intervention and the limitations on Google Analytics tracking metrics. Unique QR codes were used where possible; however, these data alone do not provide a full picture of the impact of our dissemination strategies. In addition, concurrent dissemination activities beyond this study may have brought website users to this study as well that we cannot specifically identify. Finally, effectiveness was not within the scope of this evaluation component of the study. This research gap will be addressed in our upcoming study to validate a self-management measurement tool to support an evaluation of effectiveness.

### Conclusions

Our study demonstrates that there is a need and readiness to implement a patient-facing, tailored tool to support self-management for patients experiencing early-stage CKD. Early understanding of factors to tailor implementation strategies can support successful adoption and sustainability by health care providers. Across primary care and nephrology clinics, participants believed My Kidneys My Health can support patient education and impact patient empowerment. To ensure the sustainability of this intervention, we continue to strategically disseminate to audiences within the primary and specialty care settings, support organizations to embed My Kidneys My Health into existing practices, and improve accessibility of the website by enhancing and adapting not only implementation strategies but also the website content.

## Supplementary material

10.2196/71832 Multimedia Appendix 1 Logic model.

10.2196/71832 Multimedia Appendix 2 Step 1 Interview guide.

10.2196/71832 Multimedia Appendix 3 Step 3 interview guide.

10.2196/71832 Checklist 1 COREQ checklist.

## Abbreviations

AACTT: Action, Actor, Context, Target, Time
APEASE: Acceptability, Practicability, Effectiveness, Affordability, Side effects, and Equity
Can-SOLVE CKD: Canadians Seeking Solutions and Innovations to Overcome Chronic Kidney Disease
CFIR: Consolidated Framework for Implementation Research
CKD: chronic kidney disease
COM-B: Capability Opportunity Motivation Behavior System Framework
COREQ: Consolidated Criteria for Reporting Qualitative Research
RE-AIM: Reach, Effectiveness, Adoption, Implementation, and Maintenance
TDF: Theoretical Domains Framework
