# Supplementary material for: Implementation and Evaluation of a Patient-Focused eHealth Intervention, My Kidneys My Health, in Primary Care and General Nephrology Clinics: Multimethods Study
Source: J Med Internet Res. 2025 Aug 29;27:e71832. doi: 10.2196/71832 (PMC12396798; doi:10.2196/71832)
Supplement: Multimedia Appendix 1 [file jmir-v27-e71832-s001.docx]

**Multimedia Appendix 1. Logic Model**

| **Situation** | | | | | **Priorities** | |
| --- | --- | --- | --- | --- | --- | --- |
| - Patients with early-stage chronic kidney disease (CKD) often rely on self-management (SM) and do not always have access to specialists to support care - Gap in SM resources for patients with early-stage CKD/caregivers | | | | | - Patient-oriented - Feasibility - Evidence-based | |
|  | | | | | | |
| **Program planning** | | **Program implementation** | | **Program maintenance** | | |
| **Inputs ⇨** | **Activities ⇨** | **Outputs ⇨** | **Outcomes ⇨**  ***Short term*** | **Outcomes ⇨**  ***Intermediate*** | | **Outcomes**  ***Long term*** |
| - Staff (deliver intervention) - Staff (support delivery) - Internet access - Computer access - Printer - *My Kidneys My Health* website | - Website changes - Printable materials - QR code tracking via Google Analytics - Educational materials - Learning sessions | - # Clinicians implementing - Patients reached (description) - # Resources/ strategies used or shared - # Attendees at sessions - Website users from QR code traffic sources | Increased provider:   - Awareness of website - Knowledge of website content - Ability to share website | Providers:   - Appropriately refer patients to website (who, when, how) - Regularly share website | | - Sustained integration - Change in policies (removal of barriers to sharing website) |
|  |  |  | Patients:   - Awareness of website | Patients:   - Increase use of website - Improved knowledge SM - Self-efficacy SM - Skills SM | | - Improved patient health outcomes - Prevention of disease progression |
|  | | | | | | |
| **⇧ Assumptions ⇩** | | | **⇧ External Factors ⇩** | | | |
| Behaviour change theory, RE-AIM framework | | | COVID-19 impacts service delivery, format, etc. | | | |

# 
