# Supplementary material for: Implementation and Evaluation of a Patient-Focused eHealth Intervention, My Kidneys My Health, in Primary Care and General Nephrology Clinics: Multimethods Study
Source: J Med Internet Res. 2025 Aug 29;27:e71832. doi: 10.2196/71832 (PMC12396798; doi:10.2196/71832)
Supplement: Multimedia Appendix 2 [file jmir-v27-e71832-s002.docx]

**Multimedia Appendix 2. Step 1 Interview Guide – pre-implementation**

| **Introduction** | |
| --- | --- |
| Tell me about your role around early CKD/non-dialysis patients… | - Do you make decisions on process? - How do you engage with patients? - How familiar are you with MKMH? |
| **Fit (compatibility, tension for change, adaptations, and relative priority)** | |
| What are your thoughts about the need for this website? | - How do you think it supports clinicians? - Who would it most support (i.e., which clinical roles)? |
| How does the intervention fit with existing workflow and current clinic practices? | - How would you use the website in clinic? - How might it replace/complement process/priorities? |
| How does the intervention fit your personal values and norms? | - Are there any concerns that would impede you from sharing the website? |
| How does the intervention fit with clinic’s values and norms? | - Any concerns that would impede others? - Is there technical ability to share the website? |
| What could be modified to help integrate the website into the clinic practices/fit? | - What are the likely concerns that may arise? Why? |
| **Available Resources/Support** | |
| Tell me about the resources/supports that you currently have in place to administer the website. | - Are the resources sufficient? What might help implementation? - Who will support the clinicians/staff to implement? - What shortfalls, if any, might you anticipate? |
| **Leadership Engagement** | |
| What kind of support or actions can you expect from senior leaders in your clinic to help improve the likelihood of a successful implementation? | - How do attitudes of leaders towards change vary? - What kind of support can you expect going forward? - What types of barriers might they create? |
| **Culture of Improvement and Learning** | |
| To what extent do you feel that you can try new things to improve the care or work processes? | |
| To what extent do you feel the clinic can try new things? | |
| **Communication and Collaboration** | |
| With respect to the working relationships between staff members:   - - Do staff work together collaboratively? Please explain.   - Have they established trusting relationships? Please explain.   - How is information communicated? | |
| **Sustainability** | |
| Do you feel other staff should be involved in implementation? | - Which staff? - How should they be involved? |
| How will new staff become familiar with the intervention? | - How are they familiarized with these resources? Do you feel like it’s effective? Why/why not? |
| What would keep you implementing? | - Why? How would you sustain this? |
