# Supplementary material for: Implementation and Evaluation of a Patient-Focused eHealth Intervention, My Kidneys My Health, in Primary Care and General Nephrology Clinics: Multimethods Study
Source: J Med Internet Res. 2025 Aug 29;27:e71832. doi: 10.2196/71832 (PMC12396798; doi:10.2196/71832)
Supplement: Multimedia Appendix 3 [file jmir-v27-e71832-s003.docx]

**Multimedia Appendix 3. Step 3 Interview Guide – post-implementation**

| **Introduction** | |
| --- | --- |
| Tell me about your role around early CKD/non-dialysis patients… | - Do you make decisions on process? - How do you engage with patients? - How familiar are you with MKMH? |
| **Reach** | |
| How did you hear about the My Kidneys My website? | - Through the study (completed pre-implementation interview); staff (during implementation) |
| When you learned about the website, were you interested in sharing it with patients? | - Why? |
| What made you interested in promoting the website to your patients? | - Probes: website content, access, etc.; resources/time - What made you not interested? |
| **Adoption** | |
| Have you shared the website? Who did you share it with? | - To what degree did you promote the website? (e.g., frequency, assumed reach) |
| How did you integrate the website into your clinical practice? | - What strategies worked? Why? - What strategies didn’t work? Why? |
| What made it easier to promote the website? | - Why? |
| What made it harder to promote the website? | - Which languages do you see requested? - What would be helpful content to have to access? |
| **Implementation** | |
| Are you aware of some of the strategies shared? Tell me about how you have used these? | - What do you like/not like? Why? - Have you sought other resources? Why/why not? - How did your patients react to these strategies? |
| Who do you feel this website is for? | - Informal criteria for selecting patients |
| Have you provided the website to patients that do not have CKD? | - If yes, please describe those scenarios and why you provided the website. |
| **Maintenance** | |
| What is the likelihood that you will continue to support this intervention? | - Why? |
| What would be needed for others to integrate this into clinical care? | - Effective ways to disseminate? |
| How have you changed your practice to include the website? | - What are the impacts? |
| Have you seen any changes in patient self-management? | - Diet? Medications? Using website? More confidence? Adherence? |
